# *UGT1A1* Allele Test Not Only Minimizes the Toxicity But Also Maximizes the Therapeutic Effect of Irinotecan in the Treatment of Colorectal Cancer: A Narrative Review

**DOI:** 10.3389/fonc.2022.854478

**Published:** 2022-03-09

**Authors:** Yaoyuan Li, Honggang Zheng, Xiwen Zhang, Yupeng Xi, Mengqi Cheng, Yuwei Zhao, Liya Wang, Baojin Hua

**Affiliations:** Department of Oncology, Guang’anmen Hospital, China Academy of Chinese Medical Sciences, Beijing, China

**Keywords:** *UGT1A1*, irinotecan, colorectal cancer, systematic review, precision medicine

## Abstract

**Background:**

Irinotecan is a first-line agent in the systematic treatment of colorectal cancer (CRC). Adjusting the dose of irinotecan according to the *uridine diphosphate glucuronosyltransferase* (*UGT*) *1A1* genotype reflects the principle of individualized and precision medicine, and may improve the chemotherapy response and survival of CRC.

**Methods:**

To summarize the feasibility, efficacy and safety of high dose irinotecan in CRC patients with *UGT1A1* wild-type or heterozygous alleles, PubMed, EMBASE, MEDLINE and the Cochrane Central Register of Controlled Trials online databases were searched from the date of creation to October 22, 2021.

**Results:**

A total of 1,186 related literatures were searched, and 14 studies were included for review according to the inclusion criteria. The results indicated that the maximum tolerated dose of irinotecan in CRC patients with *UGT1A1* wild-type or heterozygous variant was significantly higher than the conventional recommended dose. Chemotherapy based on high dose irinotecan improved the clinical efficacy in mCRC patients with UGT1A1*28 wild-type and heterozygous variant, and the toxicity was tolerated, as reflected in most studies.

**Conclusions:**

We are optimistic about the application of high dose irinotecan for mCRC patients with UGT1A1*28 wild-type or heterozygous variant, which will provide a relatively clear direction for future research and certain norms for clinical practice.

## Introduction

With high morbidity and mortality, colorectal cancer (CRC) is still one of the major diseases threatening human health (1, 2). In 2020, the global incidence of CRC was about 1.88 million, ranking third in the incidence of common cancer, with more than 900 thousand deaths, making it the second leading cause of cancer deaths (3). In the new diagnosis of CRC, 20% of patients have metastatic disease, and the other 25% of patients with local disease will have metastasis later (4). Irinotecan is one of the first-line agents in the systematic therapy of metastatic colorectal cancer (mCRC) (5), but its high toxicity, including severe neutropenia and diarrhea (6), has become the focus of concern in clinical use.

In recent years, the potential of pharmacogenetics in the treatment of malignant tumors has been fully reflected, which provides strong guidance for the rational application of antitumor drugs (7). *Uridine diphosphate glucuronosyltransferase* (*UGT*) *1A1* is a key enzyme for metabolism of the active metabolite SN-38 (8) of irinotecan. Studies have confirmed that *UGT1A1* gene polymorphism, with defective alleles *28 and *6 as research hotspots, affects the metabolism of irinotecan and enhances its toxicity (9–11). In clinical practice, it has been agreed to reduce the dose of irinotecan in individuals homozygous and double heterozygous mutations for UGT1A1 *28 or *6 alleles (*28/*28 or *6/*6 or *6/*28) to minimize their toxicity (12), but such treatment may be associated with poorer survival (13, 14).

Compared with patients with *UGT1A1* homozygous defect, patients with wild-type or heterozygous alleles are more efficient in metabolizing SN-38, which indicates that they may be able to tolerate the treatment of irinotecan beyond the conventional dose, resulting in better clinical outcomes. However, there seems to be no consensus on the dosage of irinotecan in patients with *UGT1A1* wild-type (*1/*1) or heterozygous alleles (such as *1/*28 or *1/*6) (15, 16). In the context of precision and personalized medicine, this is a more reasonable hypothesis. In this study, we systematically reviewed the use of high dose irinotecan in mCRC patients with *UGT1A1* wild-type or heterozygous variant to determine whether this was feasible and brought good clinical benefits and acceptable toxicity.

## Methods

### Search Strategy

We systematically searched PubMed, EMBASE, MEDLINE, and the Cochrane Central Register of Controlled Trials (CENTRAL) online databases for the period from the time of their inception to October 22, 2021. We targeted studies that selected irinotecan doses in mCRC patients based on *UGT1A1* genotype, without restrictions related to region, age or gender. We used Medical Subject Heading (MeSH) terms in PubMed, MEDLINE and CENTRAL, EMTREE terms in EMBASE. We performed keyword searches in the above four databases, and exploded them in EMBASE, MEDLINE, and CENTRAL. The search strategy is provided in the **Appendix**.

### Inclusion and Exclusion Criteria

The pieces of literature that met the inclusion criteria were those clinical studies that used higher dose of irinotecan in mCRC patients with *UGT1A1* wild-type or heterozygous variant and identified specific doses and outcomes for different genotypes. The exclusion criteria include: 1. Only different genotypes results of *UGT1A1* in the population were mentioned, but treatment results were not listed according to genotypes; 2. The sample size of the study was <10; 3. The specific dose of irinotecan in different genotypes of *UGT1A1* was not clear or the dose in each genotype was not uniform in the retrospective study; 4. The study only reflected the predictive function of *UGT1A1* polymorphism on irinotecan toxicity, and there was no specific dosage record; and 5. The study was on the toxicity or therapeutic responses of conventional doses of irinotecan.

### Study Selection and Data Collection

We used the search strategy described above to obtain relevant studies, and then selected pieces of literature based on the inclusion and exclusion criteria. Firstly, we performed a preliminary screening by using the title and abstract of a study to exclude obviously unrelated literature. Then, we downloaded the full text of the candidate papers, and finally the clinical studies of high dose irinotecan in the treatment for mCRC patients with *UGT1AI* wild-type or heterozygous variant were selected in strict accordance with the screening criteria. Two reviewers independently completed the above screening. For any discrepancy, all reviewers negotiated to reach a consensus.

Two independent reviewers used the data extraction table tested in a pilot study to extract data. If necessary, all reviewers jointly decided whether to include the data. For each included study, the main data extracted were author, year of publication, tumor stage, number of evaluable cases, treatment, recruitment period, duration of follow-up and outcome. The main outcomes included the maximum tolerated dose (MTD) of irinotecan and the therapeutic efficacy and toxicity of high dose irinotecan in mCRC patients with target genotypes. We have excluded data that were not clearly reflected in the papers.

### Data Analysis

Detailed meta-analysis was not possible due to the wide variation in the included studies. Therefore, a descriptive approach was used to summarize the data.

## Results

**Figure 1:**
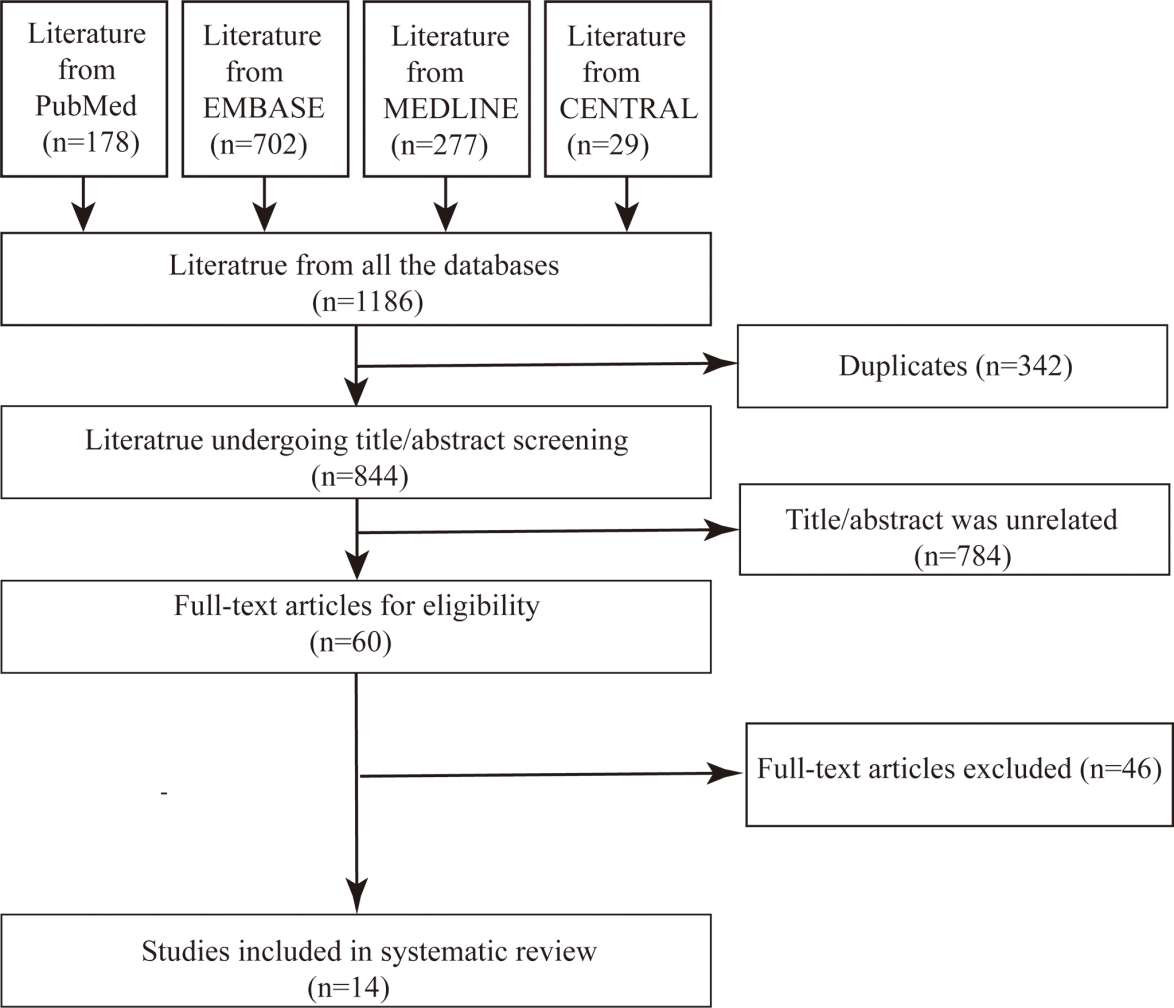
Flow diagram detailing the search strategy and results.

From the four databases, we obtained 1,186 relevant literatures. The software excluded 342 duplicates, and we identified 784 apparently unrelated studies by viewing the title and abstract. The full text was reviewed for the remaining 60 pieces of literature, excluding reviews, pieces of literature with repeated contents, and studies with unclear or low dose of irinotecan in mCRC patients with target genotypes. Finally, 14 studies were included for systematic review and summary analysis (**Figure 1**).

The included studies, from a variety of countries and ethnicities, mainly reflected the MTD of irinotecan in mCRC patients with *UGT1A1* wild-type or heterozygous variant, and the chemotherapy response, survival, and toxicity in patients with target genotypes treated with high dose irinotecan.

### MTD of Irinotecan in mCRC Patients With *UGT1A1* Wild-Type or Heterozygous Variant

Five phase I clinical trials in Asia, Europe and the United States specifically described the MTD of irinotecan in mCRC patients with *UGT1A1* wild-type and heterozygous variant (**Table 1**) (17–21). *UGT1A1* heterozygous variant in two studies from Asian populations included *1/*28 or *1/*6 (19, 20), and the other three studies only included genotype *1/*28 (17, 18, 21). In these five studies, the definition of dose limiting toxicity (DLT) was the same, i.e., hematologic grade 4 toxicity or nonhematologic grade 3 to 4 toxicity, but the definition of MTD generated from DLT was slightly different. The definition of recommended dose in two studies was closer to the conventional definition of MTD (19, 20). Therefore, we proposed to replace MTD with the recommended dose in these two studies.

**Table 1:** MTD of irinotecan in mCRC patients with *UGT1A1* wild-type or heterozygous variant.

Source	Country	Tumor stage	No. of person	Treatment	Judgment criteria	Outcome
**Toffoli et al. (** 17 **)**	Italy and US	mCRC	*1/*1, 35; *1/*28, 24	Dose adjusted irinotecan, day 1; leucovorin, 200 mg/m^2^, day 1; 5-FU at 400 mg/m^2^ bolus injection, day 1, followed by 2,400 mg/m^2^ infusion. Repeat every 2 weeks	DLT was defined as hematologic grade 4 toxicity or nonhematologic grade 3–4 toxicity. MTD was defined as the highest dose at which fewer than 2 of 10 patients experienced DLT	The MTD was 370 mg/m^2^ for *1/*1 patients and 310 mg/m^2^ for *1/*28 patients
**Marcuello et al. (** 18 **)**	Spain	White mCRC or a locally advanced recurrence after surgery	*1/*1, 42; *1/*28, 38; *28/*28, 14	Dose adjusted irinotecan, day 1; leucovorin, 200 mg/m^2^, day 1; 5-FU at 400 mg/m^2^ bolus injection, day 1, followed by 600 mg/m^2^/day×2 days infusion. Repeat every 2 weeks	DLT was defined as hematologic grade 4 toxicity or nonhematologic grade 3–4 toxicity. If 2 out of 3 or 2 out of 6 patients experienced DLT, the level below was considered MTD	The MTD was 390 mg/m^2^ for *1/*1 patients and 340 mg/m^2^ for *1/*28 patients and 130 mg/m^2^ for *28/*28 patients
**Kim et al. (** 19 **)**	Korea	mCRC	0 DA group: *1/*1, 23; 1 DA group: *1/*28 or *1/*6, 20; 2 DA group: *28/*28, *6/*6, or *28/*6, 7	Dose adjusted irinotecan plus capecitabine (2,000 mg/m^2^ days 2–15). Repeat every 3 weeks	DLT was defined as hematologic grade 4 toxicity or nonhematologic grade 3–4 toxicity. MTD was defined as DLT in 2 or more of 6 patients. The recommended dose was defined as the level below this cutoff	The recommended dose was 350 mg/m^2^, 350 mg/m^2^ and 200 mg/m^2^ f for 0, 1, 2 DA group patients, respectively
**Kim et al. (** 20 **)**	Korea	mCRC	0 DA group: *1/*1, 19; 1 DA group: *1/*28 or *1/*6, 20; 2 DA group: *28/*28, *6/*6, or *28/*6, 4	Leucovorin, 200 mg/m^2^, day 1; 5-FU at 400 mg/m^2^ bolus injection, day 1, continuous infusion of 2,400 mg/m^2^ for 46 h, followed by dose adjusted irinotecan. Repeat every 2 weeks	DLT was defined as hematologic grade 4 toxicity or nonhematologic grade 3–4 toxicity. MTD was defined as the dose level at which 2 or more of the 3–6 patients developed DLTs. The recommended dose was defined as 1 dose level under the MTD	The recommended dose was 300, 270, 150 mg/m^2^ for 0, 1, 2 DA group patients, respectively
**Toffoli et al. (** 21 **)**	US and Italy	Previously untreated mCRC	*1/*1, 25; *1/*28, 23	Dose adjusted irinotecan, day 1; leucovorin, 200 mg/m^2^, day 1; 5-FU at 400 mg/m^2^ bolus injection, day 1, followed by 2,400 mg/m^2^ infusion for 46 h; bevacizumab 5 mg/kg, day 1. Repeat every 2 weeks	DLT was defined as hematologic grade 4 toxicity or nonhematologic grade 3–4 toxicity. MTD was defined as the highest dose at which less than 4 of 10 patients had a DLT	The MTD was 310 mg/m^2^ for *1/*1 patients and 260 mg/m^2^ for *1/*28 patients

mCRC, metastatic colorectal cancer; DLT, dose-limiting toxicity; MTD, maximum tolerated dose; DA, defective allele.

In general, in the two-week regime of irinotecan combined with 5-FU and leucovorin (FOLFIRI) (17, 18, 20, 21), the MTD of irinotecan in mCRC patients with *UGT1A1* wild-type and heterozygous variant was at least greater than 260 mg/m^2^, which was significantly higher than the clinical routine dose of 180 mg/m^2^. In this study, 260 mg/m^2^ was used as the high dose cutoff value of irinotecan in FOLFIRI. Even the combination of biological agent bevacizumab did not change the pharmacokinetics of irinotecan, and its MTD was not significantly affected in the regimen of FOLFIRI combined with bevacizumab (21). In the three-week regimen combined with capecitabine, it was suggested that the TMD of irinotecan in mCRC patients with *UGT1A1* wild-type and heterozygous variant was 350 mg/m^2^, which was also significantly higher than the clinical routine dose (22).

### Outcome of High Dose Irinotecan in mCRC Patients With *UGT1A1* Wild-Type or Heterozygous Variant

There were five single-arm (23–27) trials and four double-arm trials (28–31) on the application of FOLFIRI regimen containing high dose irinotecan under the guidance of genotypes in mCRC patients with UGT1A1 *1/*1 or *1/*28. The characteristics and results of these studies are shown in **Tables 2**, **3**, respectively. The dose of irinotecan was progressively escalated in some studies, and in others the initial dose was ≥260 mg/m^2^.

**Table 2:** Single-arm Trials of High dose Irinotecan based Regimen in mCRC.

Source	Region	Study type	Tumor stage	No. of person	Treatment	Chemotherapy response	Survival time	Toxicity
**Hebbar et al. (** 23 **)**	Europe	Prospective	mCRC with metastases confined to the liver but considered to be unresectable	*1/*1 and *1/*28, 18	HD-FOLFIRI (irinotecan, 260 mg/m^2^), every 2 weeks. The feasibility of local therapy (surgery and/or radiofrequency) was assessed every 4 cycles of chemotherapy	PR, 8 (44%); SD, 6 (33.3%); DCR, 14, (77.3%); PD, 4 (22%). Local treatment was performed in 6 patients and all were complete clearance	PFS, 15.3 m; OS, 33.7 m	No grade 4 toxicity, and grade 3, 3 times
**Lu et al. (** 24 **)**	Asia	Retrospective	mCRC	Group 1,*1/*1 or *1/*28, 65; Group 2,*28/*28, 5	Bevacizumab plus FOLFIRI, every 2 weeks. Starting dose of irinotecan, 180 mg/m^2^ for Group 1 and 120 mg/m^2^ for Group 2. If toxicity ≤grade 3, the dose was escalated by 20 to 30 mg/m^2^ every 2 cycles. The maximal dose was 260, 240, and 210 mg/m^2^ for *1/*1, *1/*28 and *28/*28, respectively	Group 1: CR + PR, 50 (76.9%); SD, 11(16.9%); DCR, 61 (93.8%); PD, 4 (6.2%); Group 2: CR + PR, 1 (20%); SD, 1(20%); DCR, 2 (40%); PD, 3 (60%)	PFS was significantly different for the different *UGT1A1* genotypes (P = 0.002), and the first Group 1 was longer	Grade 3/4: Group 1, 4 patients; Group 2, 3 patients
**Manfredi et al. (** 25 **)**	Europe	Prospective	mCRC that had not previously been treated	*1/*1, 40; *1/*28, 46	Bevacizumab plus FOLFIRI, and the dose of irinotecan was 260 mg/m². The treatment was stopped in the event of patient withdrawal, disease progression, or unacceptable toxic effects	ORR: *1/*1, 18 (45%); *1/*28, 26 (56.5%)	PFS: *1/*1, 10.7 m; *1/*28, 10.4 m; OS: *1/*1, 25.5 m; *1/*28, 23.9 m	*1/*1, 30 and *1/*28, 37 exhibited at least one grade 3–4 toxicity
**Phelip et al. (** 26 **)**	Europe	Prospective	mCRC with initially borderline-resectable liver metastases, no more than 2 potentially resectable extrahepatic metastases, and *KRAS* status is wild-type	*1/*1, 9; *1/*28, 14; *28/*28, 3	High dose FOLFIRI (irinotecan 220 mg/m^2^ for *28/*28, and 260 mg/m^2^ for *1/*1 and *1/*28) plus cetuximab. Treatment was carried out for 6 cycles in the absence of disease progression or unacceptable toxicity. In cases of objective response and disease that remained unresectable, 2 more cycles were permitted. 23 patients received at least 6 cycles, of which 4 received 8 cycles	The overall cumulative 0RR at 8 cycles was 76.9% (20/26), no PD. Among the 23 patients who received at least 6 cycles, the ORR was 82.6%, metastasectomy, 21 (80.7%)	PFS, 15.8 m, 3-year PFS was 23.3%; OS was not reached and 3-year OS was 66.1%. Among 21 resected patients, 18 (85.7%) had a relapse, with a median relapse-free survival of 15.3 m	Grade 3–4 toxicity: neutropenia (31%), diarrhea (20.8%), anorexia (16.4%). No deaths due to toxicity
**Ma et al. (** 27 **)**	Asia	Prospective	mCRC who were previously treated with FOLFOX, FOLFIRI, anti-VEGFR, or anti-EGFR if *KRAS* was wild-type	*1/*1, 12; *1/*28, 0; *28/*28, 1	Regorafenib plus FOLFIRI with irinotecan dose escalation (starting dose was 180 mg/m^2^ for *1/*1 and 120 mg/m^2^ for *28/*28). The dose was increased by 30 mg/m^2^ every two cycles until grade ≥3 AEs. The highest prescribed irinotecan dose was 290 mg/m^2^ for *1/*1 and 120 mg/m^2^ for *28/*28	PR, 2 (15.4%); SD, 7 (53.8%); DCR, (69.2%); PD, 4 (30.8%)	PFS, 9.5 m (95% CI: 3.0–16.0); OS, 13.0 m (95% CI: 7.2–18.8)	Grade ≥3 AE: hand–foot syndrome, 8; mucositis, 5; neutropenia, 4; diarrhea, 4; fatigue, 3

mCRC, metastatic colorectal cancer; CR, complete response; PR, partial response; ORR, objective response rate; SD, stable disease; DCR, disease control rate; PD, progressive disease; PFS, progression-free survival; OS, overall survival; AE, adverse event; FOLFOX, Oxaliplatin combined with 5-FU and leucovorin; FOLFIRI, irinotecan combined with 5-FU and leucovorin; VEGFR, vascular epithelial growth factor receptor; EGFR, epidermal growth factor receptor; CI, confidence interval.

**Table 3:** Double-arm Trials of High dose Irinotecan based Regimen in mCRC.

Source	Region	Study type	Tumor stage	No. of person	Treatment	Chemotherapy response	Survival time	Toxicity
**Lu et al. (** 28 **)**	Asia	Retrospective	mCRC	HDG, 79; RDG, 28	Bevacizumab plus FOLFIRI. Starting dose of irinotecan, 180 mg/m^2^ for *1/*1 and *1/*28, 120 mg/m^2^ for *28/*28 in HDG. If toxicity ≤grade 3, the dose was escalated by 30 mg/m^2^. The maximal dose was 260, 240, 210 mg/m^2^ for *1/*1, *1/*28, and *28/*28, respectively. The dose of RDG was 180 mg/m^2^	ORR, 55 (69.6%) and 13 (46.4%); SD + PD, 24 (30.4%) and 15 (53.6%) in HDG and RDG, respectively (p = 0.028)	PFS, 12.2 m and 9.4 m in HDG and RDG, respectively (P = 0.025); OS, NA	Grade 3/4 AEs were not significantly different between the 2 groups (P = 0.189)
**Páez et al. (** 29 **)**	Europe	Randomized, multicenter, open-label, non-blinded	mCRC with UGT1A1 *1/*1 or *1/*28, no prior treatment for metastatic disease	HDG, 40; RDG, 39	FOLFIRI. Irinotecan doses for *1/*1 and *1/*28 in HDG were 300 and 260 mg/m^2^, respectively; and 180 mg/m^2^ was administered in RDG	ORR, 27 (67.5%) and 17 (43.6%) (p = 0.001); SD, 3 (7.5%) and 17 (43.6%); PD, 10 (25%) and 5 (12.8%); metastasectomy, 9 (22.5%) and 6 (15.4%) in HDG and RDG, respectively	PFS, 8.6 m and 8.2 m (P = 0.46); OS, 26 m and 17.6 m (P = 0.74) in HDG and RDG, respectively	No significant differences in grade 3–4 toxicities between the two groups. No differences in serious AEs, dose reduction, or use of G-CSF
**Tsai et al. (** 30 **)**	Asia	Multicenter, randomized, controlled, open-label	mCRC	HDG: *1/*1, 82; *1/*28, 23; *28/*28, 2; RDG,106	Bevacizumab plus FOLFIRI. Starting dose of irinotecan, 180 mg/m^2^ for *1/*1 and *1/*28, 120 mg/m^2^ for *28/*28 in HDG. If toxicity ≤grade 3, the dose was escalated by 30 mg/m^2^. The maximal dose was 260, 240, 180 mg/m^2^ for *1/*1, *1/*28, and *28/*28, respectively. The dose of RDG was 180 mg/m^2^	ORR, 77 (71.9%) and 44 (41.5%) (p <0.001); DCR, 98 (91.6%) and 83 (78.2%) (p = 0.007); PD, 9 (8.4%) and 23 (21.7%); metastasectomy, 34 (31.8%) and 17 (16.1%) (p = 0.007) in HDG and RDG, respectively	PFS, 14.0 m and 10.0 m (P <0.001); OS, 30 m and 22 m (P = 0.02) in HDG and RDG, respectively	Irinotecan-related grade 3/4 AEs, 25 (23.4%) and 25 (23.6%) in HDG and RDG, respectively (P = 0.520)
**Hsieh et al. (** 31 **)**	Asia	Retrospective	mCRC with *BRAF* mutation, and UGT1A1 *1/*1 or *1/*28	HDG, 8; RDG, 9	Bevacizumab plus FOLFIRI. Starting dose of irinotecan was 180 mg/m^2^ and the dose was escalated 20–30 mg/m^2^ until grade 3/4 AEs in HDG. The maximum dose reached 260, 240, 210 mg/m^2^ in 4, 2 and 2 patients, respectively. The dose of RDG was 180 mg/m^2^	PR, 1 (12.5%) and 1 (11.1%); SD, 5 (62.5%) and 4 (44.4%); DCR, 75% and 55.6%; PD, 2 (25%) and 4 (44.4%) in HDG and RDG, respectively (p = 0.697)	PFS, 11.5 m and 5.7 m (P = 0.552); OS, 15.8 m and 14.5 m (P = 0.40) in HDG and RDG, respectively	In total, grade 3 toxicity, 2 (11.8%) patients, and no grade 4/5 toxicity

mCRC, metastatic colorectal cancer; HDG, High dose group; RDG, routine dose group; FOLFIRI, irinotecan combined with 5-FU and leucovorin; ORR, objective response rate; SD, stable disease; DCR, disease control rate; PD, progressive disease; PFS, progression-free survival; OS, overall survival; AE, adverse event.

### Single-Arm Trials Results

In the single-arm trials, three studies from Europe directly used high dose irinotecan according to the guidance of *UGT1A1* genotypes (23, 25, 26), while two studies from Asia used the dose escalation of irinotecan (24, 27).

A prospective study was about high dose FOLFIRI (260 mg/m^2^) combined with local surgery and radiofrequency ablation for the unresectable liver metastases from CRC patients with UGT1A1 *1/*1 or *1/*28 (23). Due to insufficient recruitment, the trial was terminated early. The evaluable results showed good objective response rate (ORR), disease control rate (DCR), high complete clearance rate, promising survival and excellent safety.

Three studies focused on high dose FOLFIRI in combination with biologics for mCRC. One of these, direct high dose FOLFIRI plus bevacizumab, was prematurely terminated due to the high incidence of overly strict toxicity events specified in the trial protocol, leading to a possible outcome bias (25). The other two studies were dose-escalating FOLFIRI combined with bevacizumab (24) and direct high dose FOLFIRI combined with cetuximab (26) based on *UGT1A1* genotypes, including, *UGT1A1* homozygous variant (*28/*28). The results suggested that high dose FOLFIRI combined with biological agents can have good chemotherapy response rate, survival and tolerable toxicity in mCRC with *UGT1A1* wild-type and heterozygous variant.

Another trial of dose-escalating FOLFIRI in combination with regorafenib based on *UGT1A1* genotypes, as a non-first-line treatment for mCRC, also demonstrated a tolerable toxicity associated with irinotecan and an effective clinical outcome that can improve survival (27).

### Double-Arm Trials Results

In the double-arm trials, both retrospective and prospective studies demonstrated similar safety and no significant difference in toxicity between high dose and routine dose FOLFIRI based on *UGT1A1* genotypes in mCRC.

In the two retrospective studies, the treatment was FOLFIRI combined with bevacizumab. In the high dose group of mCRC with UGT1A1 *1/*1 or *1/*28, the dose of irinotecan was gradually increased to or close to 260 mg/m^2^, while the dose of irinotecan in the routine dose group remained at 180 mg/m^2^. One of the studies showed statistically significant improvements in chemotherapy response (p = 0.028) and progression-free survival (PFS) (p = 0.025) in the high dose group over the routine dose group (28). In another small sample study of mCRC with *BRAF* mutation, although the high dose group showed an advantage in survival, there was no statistically significant difference (31).

A multicenter, open-label randomized trial from Europe (29) showed that in mCRC with UGT1A1 *1/*1 or *1/*28, high dose FOLFIRI group (irinotecan doses was 300 mg/m^2^ for *1/*1 and 260 mg/m^2^ for *1/*28) had a significant advantage in chemotherapy response (p = 0.001). The survival was also better than that in the routine dose group, but was not statistically significant.

In a multicenter, open-label randomized controlled trial from Asia (30), the high dose group was treated with dose-escalating FOLFIRI combined with bevacizumab for mCRC and the maximal dose of irinotecan was 260, 240, and 180 mg/m^2^ for UGT1A1 *1/*1, *1/*28, and *28/*28, respectively. The dose of irinotecan in the routine dose group remained at 180 mg/m^2^ without *UGT1A1* test. Compared with the routine dose group, the high dose group showed significant advantages in chemotherapy response (ORR, P <0.001; DCR, p = 0.007; metastasectomy, p = 0.007) and survival (PFS, P <0.001; OS, P = 0.02).

## Discussion

Previous systematic review suggested that for low dose irinotecan, the absolute risk of toxicity in patients with UGT1A1 *28/*28 genotype is similar to the overall risk of all patients. However, moderate and high dose irinotecan increased the absolute risk of toxicity (32, 33), suggesting the tolerance of patients with *UGT1A1* wild-type or heterozygous variant to high dose of irinotecan. With conventional dose of irinotecan, the chemotherapy response rate and survival of patients with UGT1A1 *1/*1 was worse than that of patients with UGT1A1 *28/*28 (34–36), which was associated with the higher glucuronidation rate of SN38 (34). Therefore, in order to improve the response rate and even survival of chemotherapy based on irinotecan in patients with *UGT1A1* wild-type or heterozygous variant, it is necessary to reasonably increase the dose of irinotecan on the premise of tolerable toxicity. The results of this systematic review basically confirm this hypothesis.

The studies finally included in this system review have been carried out in the past decade, from the exploration of the MTD of irinotecan in CRC patients with *UGT1A1* wild-type and heterozygous variant in the early stage, to the single-arm study of the therapeutic effect of high dose irinotecan on target patient groups, and to the randomized controlled double-arm trials carried out in recent years. These studies have shown that the MTD of irinotecan in CRC patients with *UGT1A1* wild-type and heterozygous variant was significantly higher than the conventional recommended dose. Chemotherapy based on high dose irinotecan in the treatment of mCRC patients with UGT1A1 *28 wild-type and heterozygous variant can improve the clinical efficacy, and the toxicity was tolerable, which has been reflected in most studies.

In the first-line treatment, high dose FOLFIRI alone has shown a satisfactory chemotherapy response rate (23, 29), with an ORR of 67.5% in the randomized controlled study, although there was no statistical advantage in survival (29). Chemotherapy combined with biologics has shown a survival advantage over chemotherapy alone in first-line treatment of mCRC (37–40). High dose FOLFIRI in combination with bevacizumab or cetuximab in patients with wild-type or heterozygous variant of UGT1A1 *28 have yielded good clinical outcomes (24–26, 28, 30). Survival in the randomized controlled trial (30) is equivalent to that of FOLFOXIRI (fluorouracil, leucovorin, oxaliplatin, and irinotecan) combined with bevacizumab (41, 42). This makes us look forward to the study of high dose FOLFOXIRI combined with anti-vascular endothelial growth factor receptor or anti-epidermal growth factor receptor or immunotherapy in selected target mCRC patients according to *UGT1A1* genotypes.

*BRAF* mutation exists in approximately 10% of CRC (43). The *BRAF* mutation is related to reduced overall survival and poor treatment response compared with tumors with wild-type *BRAF* (44, 45). High dose FOLFIRI in mCRC patients with UGT1A1 *28 wild-type or heterozygous variant and *BRAF* mutant showed a survival advantage (31) and represented statistical difference from the routine dose in the subgroup analysis of a randomized controlled trial (29). In addition, in the small sample trial of non-first-line treatment, the exploration of adjusting the dose of irinotecan in mCRC patients based on *UGT1A1* has also achieved positive results (27). These encouraging results suggested a bright application prospect of high dose irinotecan in multi-stages and multi-genotypes of mCRC with *UGT1A1* wild-type or heterozygous variant.

In view of the available evidence and the convenience of commercial test (46), we recommend testing *UGT1A1* in CRC patients who will undergo irinotecan-based chemotherapy and increasing the dose of irinotecan in patients with UGT1A1 *28 wild-type or heterozygous variant to improve clinical efficacy, which is consistent with the Pan-Asian adapted European Society for Medical Oncology consensus guidelines (47) and the latest comments of Karas et al. (48). It can be applied directly with the recommended MTD or gradually increased from routine dose to MTD by chemotherapy cycle based on safety considerations. For patients with UGT1A1 *28/*28 mutant alleles, initial dose of irinotecan can be reduced by 30% based on safety considerations, although this may lead to poor chemotherapy response and survival (49). However, the dose escalation to standard or higher dose can be considered according to the tolerance of toxicity.

Due to the large heterogeneity of the studies included in this review, such as differences in study types, dose increase methods of irinotecan, treatment methods, types of participants, etc., the outcome could not be quantitatively pooled, but qualitative analysis, which reduced the reliability of evidence. In addition, the included clinical studies of high dose irinotecan for mCRC, whether single-arm or double-arm trials, did not cover the UGT1A1 *6 genotype, which has a greater impact on the Asian population (50–52). These were the obvious major deficiencies of this review, and it is expected that more high-quality randomized controlled studies will be conducted in the future to provide high-level evidence support for the rational application of chemotherapy based on high dose irinotecan in CRC patients with *UGT1A1* wild-type or heterozygous.

In conclusion, through the summary of this review, we are optimistic about the application of high dose irinotecan for mCRC patients with UGT1A1 *28 wild-type or heterozygous variant, which will provide a clear direction for future clinical research and certain norms for clinical practice.

## Data Availability Statement

The original contributions presented in the study are included in the article/**Supplementary Material**. Further inquiries can be directed to the corresponding author.

## Author Contributions

HZ and BH designed the project. YL, XZ, YX, and LW searched pieces of literature and wrote the manuscript. YL, YZ and MC revised the manuscript. All authors listed have made a substantial, direct, and intellectual contribution to the work and approved it for publication.

## Funding

This study was supported by the National Natural Scientific Foundation of China (Nos. 82174463 and 82174465) and the CACMS Innovation Fund (No. CI2021A01804). The funding sources had no role in the design and conduct of the study and collection, management, analysis, and interpretation of the data.

## Conflict of Interest

The authors declare that the research was conducted in the absence of any commercial or financial relationships that could be construed as a potential conflict of interest.

## Publisher’s Note

All claims expressed in this article are solely those of the authors and do not necessarily represent those of their affiliated organizations, or those of the publisher, the editors and the reviewers. Any product that may be evaluated in this article, or claim that may be made by its manufacturer, is not guaranteed or endorsed by the publisher.

## References

[1] Baidoun F Elshiwy K Elkeraie Y Merjaneh Z Khoudari G Sarmini MT . Colorectal Cancer Epidemiology: Recent Trends and Impact on Outcomes. Curr Drug Targets (2021) 22(9):998–1009. doi: 10.2174/1389450121999201117115717 33208072

[2] Aparicio J Esposito F Serrano S Falco E Escudero P Ruiz-Casado A . Metastatic Colorectal Cancer. First Line Therapy for Unresectable Disease. J Clin Med (2020) 9(12):3889. doi: 10.3390/jcm9123889 PMC7761096 33265959

[3] Sung H Ferlay J Siegel RL Laversanne M Soerjomataram I Jemal A . Global Cancer Statistics 2020: GLOBOCAN Estimates of Incidence and Mortality Worldwide for 36 Cancers in 185 Countries. CA: Cancer J Clin (2021) 71(3):209–49. doi: 10.3322/caac.21660 33538338

[4] Biller LH Schrag D . Diagnosis And Treatment of Metastatic Colorectal Cancer: A Review. Jama (2021) 325(7):669–85. doi: 10.1001/jama.2021.0106 33591350

[5] Benson AB Venook AP Al-Hawary MM Arain MA Chen YJ Ciombor KK . Colon Cancer, Version 2.2021, NCCN Clinical Practice Guidelines in Oncology. J Natl Compr Canc Netw (2021) 19(3):329–59. doi: 10.6004/jnccn.2021.0012 33724754

[6] Paulík A Nekvindová J Filip S . Irinotecan Toxicity During Treatment of Metastatic Colorectal Cancer: Focus on Pharmacogenomics and Personalized Medicine. Tumori (2020) 106(2):87–94. doi: 10.1177/0300891618811283 30514181

[7] Relling MV Evans WE . Pharmacogenomics in the Clinic. Nature (2015) 526(7573):343–50. doi: 10.1038/nature15817 PMC4711261 26469045

[8] Etienne-Grimaldi MC Boyer JC Thomas F Quaranta S Picard N Loriot MA . UGT1A1 Genotype and Irinotecan Therapy: General Review and Implementation in Routine Practice. Fundam Clin Pharmacol (2015) 29(3):219–37. doi: 10.1111/fcp.12117 25817555

[9] O’Dwyer PJ Catalano RB . Uridine Diphosphate Glucuronosyltransferase (UGT) 1A1 and Irinotecan: Practical Pharmacogenomics Arrives in Cancer Therapy. J Clin Oncol: Off J Am Soc Clin Oncol (2006) 24(28):4534–8. doi: 10.1200/jco.2006.07.3031 17008691

[10] Takano M Sugiyama T . UGT1A1 Polymorphisms in Cancer: Impact on Irinotecan Treatment. Pharmacogenomics personalized Med (2017) 10:61–8. doi: 10.2147/pgpm.s108656 PMC5338934 28280378

[11] Iyer L Hall D Das S Mortell MA Ramírez J Kim S . Phenotype-Genotype Correlation of *In Vitro* SN-38 (Active Metabolite of Irinotecan) and Bilirubin Glucuronidation in Human Liver Tissue With UGT1A1 Promoter Polymorphism. Clin Pharmacol Ther (1999) 65(5):576–82. doi: 10.1016/s0009-9236(99)70078-0 10340924

[12] Hulshof EC Deenen MJ Guchelaar HJ Gelderblom H . Pre-Therapeutic UGT1A1 Genotyping to Reduce the Risk of Irinotecan-Induced Severe Toxicity: Ready for Prime Time. Eur J Cancer (Oxf Engl: 1990) (2020) 141:9–20. doi: 10.1016/j.ejca.2020.09.007 33125947

[13] Ichikawa W Uehara K Minamimura K Tanaka C Sadahiro S Shinozaki K . Impact of UGT1A1 Genotype and Irinotecan Exposure on Outcomes in Japanese Patients With Advanced Colorectal Cancer Treated by Irinotecan-Based Regimens. Eur J Cancer (2015) 3):S362. doi: 10.1016/S0959-8049(16)31022-X

[14] Yu Q Zhang T Xie C Qiu H Liu B Huang L . UGT1A Polymorphisms Associated With Worse Outcome in Colorectal Cancer Patients Treated With Irinotecan-Based Chemotherapy. Cancer Chemother Pharmacol (2018) 82(1):87–98. doi: 10.1007/s00280-018-3595-7 29728798

[15] Nelson RS Seligson ND Bottiglieri S Carballido E Cueto AD Imanirad I . UGT1A1 Guided Cancer Therapy: Review of the Evidence and Considerations for Clinical Implementation. Cancers (2021) 13(7):1566. doi: 10.3390/cancers13071566 33805415 PMC8036652

[16] Chen X Liu L Guo Z Liang W He J Huang L . UGT1A1 Polymorphisms With Irinotecan-Induced Toxicities and Treatment Outcome in Asians With Lung Cancer: A Meta-Analysis. Cancer Chemother Pharmacol (2017) 79(6):1109–17. doi: 10.1007/s00280-017-3306-9 28502040

[17] Toffoli G Cecchin E Gasparini G D’Andrea M Azzarello G Basso U . Genotype-Driven Phase I Study of Irinotecan Administered in Combination With Fluorouracil/Leucovorin in Patients With Metastatic Colorectal Cancer. J Clin Oncol: Off J Am Soc Clin Oncol (2010) 28(5):866–71. doi: 10.1200/jco.2009.23.6125 PMC4872310 20038727

[18] Marcuello E Páez D Paré L Salazar J Sebio A del Rio E . A Genotype-Directed Phase I-IV Dose-Finding Study of Irinotecan in Combination With Fluorouracil/Leucovorin as First-Line Treatment in Advanced Colorectal Cancer. Br J Cancer (2011) 105(1):53–7. doi: 10.1038/bjc.2011.206 PMC3137420 21654688

[19] Kim KP Kim HS Sym SJ Bae KS Hong YS Chang HM . A UGT1A1*28 and *6 Genotype-Directed Phase I Dose-Escalation Trial of Irinotecan With Fixed-Dose Capecitabine in Korean Patients With Metastatic Colorectal Cancer. Cancer Chemother Pharmacol (2013) 71(6):1609–17. doi: 10.1007/s00280-013-2161-6 23595344

[20] Kim KP Hong YS Lee JL Bae KS Kim HS Shin JG . A Phase I Study of UGT1A1 *28/*6 Genotype-Directed Dosing of Irinotecan (CPT-11) in Korean Patients With Metastatic Colorectal Cancer Receiving FOLFIRI. Oncology (2015) 88(3):164–72. doi: 10.1159/000368674 25427841

[21] Toffoli G Sharma MR Marangon E Posocco B Gray E Mai Q . Genotype-Guided Dosing Study of FOLFIRI Plus Bevacizumab in Patients With Metastatic Colorectal Cancer. Clin Cancer Res: an Off J Am Assoc Cancer Res (2017) 23(4):918–24. doi: 10.1158/1078-0432.ccr-16-1012 PMC6777349 27507617

[22] Xu RH Muro K Morita S Iwasa S Han SW Wang W . Modified XELIRI (Capecitabine Plus Irinotecan) Versus FOLFIRI (Leucovorin, Fluorouracil, and Irinotecan), Both Either With or Without Bevacizumab, as Second-Line Therapy for Metastatic Colorectal Cancer (AXEPT): A Multicentre, Open-Label, Randomised, non-Inferiority, Phase 3 Trial. Lancet Oncol (2018) 19(5):660–71. doi: 10.1016/s1470-2045(18)30140-2 29555258

[23] Hebbar M Truant S Desauw C Sergent-Baudson G Cattan S Piessen G . High-Dose FOLFIRI, Surgery, and Radiofrequency Ablation for Patients With Unresectable Liver Metastases From Colorectal Cancer. Anticancer Res (2013) 33(4):1603–8. 23564804

[24] Lu CY Huang CW Wu IC Tsai HL Ma CJ Yeh YS . Clinical Implication of UGT1A1 Promoter Polymorphism for Irinotecan Dose Escalation in Metastatic Colorectal Cancer Patients Treated With Bevacizumab Combined With FOLFIRI in the First-Line Setting. Trans Oncol (2015) 8(6):474–9. doi: 10.1016/j.tranon.2015.11.002 PMC4700286 26692528

[25] Manfredi S Bouche O Rougier P Dahan L Loriot MA Aparicio T . High-Dose FOLFIRI Plus Bevacizumab in the Treatment of Metastatic Colorectal Cancer Patients With Two Different UGT1A1 Genotypes: FFCD 0504 Study. Mol Cancer Ther (2015) 14(12):2782–8. doi: 10.1158/1535-7163.MCT-15-0293 26494856

[26] Phelip JM Mineur L de la Fouchardière C Chatelut E Quesada JL Roblin X . High Resectability Rate of Initially Unresectable Colorectal Liver Metastases After UGT1A1-Adapted High-Dose Irinotecan Combined With LV5FU2 and Cetuximab: A Multicenter Phase II Study (ERBIFORT). Ann Surg Oncol (2016) 23(7):2161–6. doi: 10.1245/s10434-015-5072-4 26739304

[27] Ma CJ Huang CW Yeh YS Tsai HL Hu HM Wu IC . Regorafenib Plus FOLFIRI With Irinotecan Dose Escalated According to Uridine Diphosphate Glucuronosyltransferase 1A1 Genotyping in Patients With Metastatic Colorectal Cancer. Oncol Res (2017) 25(5):673–9. doi: 10.3727/97818823455816x14786040691928 PMC7840952 27938508

[28] Lu CY Huang CW Hu HM Tsai HL Huang CM Yu FJ . Prognostic Advantage of Irinotecan Dose Escalation According to Uridine Diphosphate Glucuronosyltransferase 1A1 (UGT1A1) Genotyping in Patients With Metastatic Colorectal Cancer Treated With Bevacizumab Combined With 5-Fluorouracil/Leucovorin With Irinotecan in a First-Line Setting. Transl Res (2014) 164(2):169–76. doi: 10.1016/j.trsl.2013.12.009 24462762

[29] Páez D Tobeña M Fernández-Plana J Sebio A Virgili AC Cirera L . Pharmacogenetic Clinical Randomised Phase II Trial to Evaluate the Efficacy and Safety of FOLFIRI With High-Dose Irinotecan (HD-FOLFIRI) in Metastatic Colorectal Cancer Patients According to Their UGT1A 1 Genotype. Br J Cancer (2019) 120(2):190–5. doi: 10.1038/s41416-018-0348-7 PMC6342907 30585257

[30] Tsai HL Huang CW Lin YW Wang JH Wu CC Sung YC . Determination of the UGT1A1 Polymorphism as Guidance for Irinotecan Dose Escalation in Metastatic Colorectal Cancer Treated With First-Line Bevacizumab and FOLFIRI (PURE FIST). Eur J Cancer (Oxford England: 1990) (2020) 138:19–29. doi: 10.1016/j.ejca.2020.05.031 32829105

[31] Hsieh YC Chang TK Su WC Huang CW Tsai HL Chen YC . UGT1A1 Polymorphism for Irinotecan Dose Escalation in Patients With BRAF -Mutated Metastatic Colorectal Cancer Treated With First-Line Bevacizumab and FOLFIRI. J Oncol (2021) 2021:6686517. doi: 10.1155/2021/6686517 33777142 PMC7972843

[32] Hoskins JM Goldberg RM Qu P Ibrahim JG Mcleod HL . UGT1A1*28 Genotype and Irinotecan-Induced Neutropenia: Dose Matters. J Natl Cancer Institute (2007) 99(17):1290–5. doi: 10.1093/jnci/djm115 17728214

[33] Hu ZY Yu Q Zhao YS . Dose-Dependent Association Between UGT1A1*28 Polymorphism and Irinotecan-Induced Diarrhoea: A Meta-Analysis. Eur J Cancer (2010) 46(10):1856–65. doi: 10.1016/j.ejca.2010.02.049 20335017

[34] Toffoli G Cecchin E Corona G Russo A Buonadonna A D’Andrea M . The Role of UGT1A1*28 Polymorphism in the Pharmacodynamics and Pharmacokinetics of Irinotecan in Patients With Metastatic Colorectal Cancer. J Clin Oncol: Off J Am Soc Clin Oncol (2006) 24(19):3061–8. doi: 10.1200/jco.2005.05.5400 16809730

[35] Kimura K Yamano T Igeta M Imada A Jihyung S Babaya A . UGT1A1 Polymorphisms in Rectal Cancer Associated With the Efficacy and Toxicity of Preoperative Chemoradiotherapy Using Irinotecan. Cancer Sci (2018) 109(12):3934–42. doi: 10.1111/cas.13807 PMC6272094 30246377

[36] Stein G Stelmach P Kasper S Paul A Wedemeyer HH Schmid KW . The Prognostic Impact of UDP-Glucuronyltransferase 1A1*1 (UGT1A1*1) Polymorphism in Patients With Advanced Colorectal and non-Colorectal Cancer Treated With Irinotecanbased Systemic Chemotherapies. Oncol Res Treat (2018) 41(Supplement 4):122–3. doi: 10.1159/000492737

[37] Heinemann V von Weikersthal LF Decker T Kiani A Vehling-Kaiser U Al-Batran SE . FOLFIRI Plus Cetuximab Versus FOLFIRI Plus Bevacizumab as First-Line Treatment for Patients With Metastatic Colorectal Cancer (FIRE-3): A Randomised, Open-Label, Phase 3 Trial. Lancet Oncol (2014) 15(10):1065–75. doi: 10.1016/s1470-2045(14)70330-4 25088940

[38] Qin S Li J Wang L Xu J Cheng Y Bai Y . Efficacy and Tolerability of First-Line Cetuximab Plus Leucovorin, Fluorouracil, and Oxaliplatin (FOLFOX-4) Versus FOLFOX-4 in Patients With RAS Wild-Type Metastatic Colorectal Cancer: The Open-Label, Randomized, Phase III TAILOR Trial. J Clin Oncol: Off J Am Soc Clin Oncol (2018) 36(30):3031–9. doi: 10.1200/jco.2018.78.3183 PMC6324088 30199311

[39] Tang W Ren L Liu T Ye Q Wei Y He G . Bevacizumab Plus Mfolfox6 Versus Mfolfox6 Alone as First-Line Treatment for RAS Mutant Unresectable Colorectal Liver-Limited Metastases: The BECOME Randomized Controlled Trial. J Clin Oncol: Off J Am Soc Clin Oncol (2020) 38(27):3175–84. doi: 10.1200/jco.20.00174 32749938

[40] Heinemann V von Weikersthal LF Decker T Kiani A Kaiser F Al-Batran SE . FOLFIRI Plus Cetuximab or Bevacizumab for Advanced Colorectal Cancer: Final Survival and Per-Protocol Analysis of FIRE-3, a Randomised Clinical Trial. Br J Cancer (2021) 124(3):587–94. doi: 10.1038/s41416-020-01140-9 PMC7851157 33154570

[41] Cremolini C Loupakis F Antoniotti C Lupi C Sensi E Lonardi S . FOLFOXIRI Plus Bevacizumab Versus FOLFIRI Plus Bevacizumab as First-Line Treatment of Patients With Metastatic Colorectal Cancer: Updated Overall Survival and Molecular Subgroup Analyses of the Open-Label, Phase 3 TRIBE Study. Lancet Oncol (2015) 16(13):1306–15. doi: 10.1016/s1470-2045(15)00122-9 26338525

[42] Loupakis F Cremolini C Masi G Lonardi S Zagonel V Salvatore L . Initial Therapy With FOLFOXIRI and Bevacizumab for Metastatic Colorectal Cancer. New Engl J Med (2014) 371(17):1609–18. doi: 10.1056/NEJMoa1403108 25337750

[43] Grothey A Fakih M Tabernero J . Management of BRAF-Mutant Metastatic Colorectal Cancer: A Review of Treatment Options and Evidence-Based Guidelines. Ann Oncol: Off J Eur Soc Med Oncol (2021) 32(8):959–67. doi: 10.1016/j.annonc.2021.03.206 33836264

[44] Morris VK Bekaii-Saab T . Improvements in Clinical Outcomes for BRAF(V600E) -Mutant Metastatic Colorectal Cancer. Clin Cancer Res: an Off J Am Assoc Cancer Res (2020) 26(17):4435–41. doi: 10.1158/1078-0432.ccr-19-3809 32253230

[45] Kanat O Ertas H . Caner B . Contemporary Treatment Approaches for Metastatic Colorectal Cancer Driven by BRAF V600 Mutations. World J Gastrointestinal Oncol (2020) 12(10):1080–90. doi: 10.4251/wjgo.v12.i10.1080 PMC7579731 33133378

[46] Van Bebber SL Keegan HL Phillips KA Issa AM . Novel Personalized Medicine Technology: UGT1A1 Testing for Irinotecan as a Case Study. Personalized Med (2006) 3(4):415–9. doi: 10.2217/17410541.3.4.415 29788595

[47] Yoshino T Arnold D Taniguchi H Pentheroudakis G Yamazaki K Xu RH . Pan-Asian Adapted ESMO Consensus Guidelines for the Management of Patients With Metastatic Colorectal Cancer: A JSMO-ESMO Initiative Endorsed by CSCO, KACO, MOS, SSO and TOS. Ann Oncol: Off J Eur Soc Med Oncol (2018) 29(1):44–70. doi: 10.1093/annonc/mdx738 29155929

[48] Karas S Innocenti F . All You Need to Know About UGT1A1 Genetic Testing for Patients Treated With Irinotecan: A Practitioner-Friendly Guide. JCO Oncol Pract (2021) OP2100624. doi: 10.1200/op.21.00624 PMC9014426 34860573

[49] Iwasa S Muro K Morita S Park YS Nakamura M Kotaka M . Impact of UGT1A1 Genotype on the Efficacy and Safety of Irinotecan-Based Chemotherapy in Metastatic Colorectal Cancer. Cancer Sci (2021) 112(11):4669–78. doi: 10.1111/cas.15092 PMC8586680 34327766

[50] Zhang X Yin JF Zhang J Kong SJ Zhang HY Chen XM . UGT1A1*6 Polymorphisms are Correlated With Irinotecan-Induced Neutropenia: A Systematic Review and Meta-Analysis. Cancer Chemother Pharmacol (2017) 80(1):135–49. doi: 10.1007/s00280-017-3344-3 28585035

[51] Hikino K Ozeki T Koido M Terao C Kamatani Y Murakami Y . Comparison of Effects of UGT1A1*6 and UGT1A1*28 on Irinotecan-Induced Adverse Reactions in the Japanese Population: Analysis of the Biobank Japan Project. J Hum Genet (2019) 64(12):1195–202. doi: 10.1038/s10038-019-0677-2 31586129

[52] Gao J Zhou J Li Y Lu M Jia R Shen L . UGT1A1 6/28 Polymorphisms Could Predict Irinotecan-Induced Severe Neutropenia Not Diarrhea in Chinese Colorectal Cancer Patients. Med Oncol (Northwood London England) (2013) 30(3):604. doi: 10.1007/s12032-013-0604-x 23686699

