# Supplementary material for: UGT1A1 Allele Test Not Only Minimizes the Toxicity But Also Maximizes the Therapeutic Effect of Irinotecan in the Treatment of Colorectal Cancer: A Narrative Review
Source: Front Oncol. 2022 Mar 9;12:854478. doi: 10.3389/fonc.2022.854478 (PMC8959381; doi:10.3389/fonc.2022.854478)
Supplement: Supplementary file 1 [file Table_1.docx]

**Search Strategy of PubMed**

**#1** **(((((Colorectal Neoplasms[MeSH Terms]) OR (Colorectal tumor?[Title/Abstract])) OR (Colorectal cancer?[Title/Abstract])) OR (Colorectal Carcinoma?[Title/Abstract])) OR ((((Colonic Neoplasms[MeSH Terms]) ) OR (Colon* cancer?[Title/Abstract])) OR (Colon* tumor?[Title/Abstract]))) OR (((Rectal Neoplasms[MeSH Terms]) OR (Rect* cancer?[Title/Abstract])) OR (Rect* tumor?[Title/Abstract])) 354253**

**#2** **((((((((Irinotecan[MeSH Terms]) OR (Irrinotecan[Title/Abstract])) OR (Camptothecin?11[Title/Abstract])) OR (SN?38?11[Title/Abstract])) OR (SN?38[Title/Abstract])) OR (NK012 Compound[Title/Abstract])) OR (CPT?11[Title/Abstract])) OR (Camptosar[Title/Abstract])) OR (7-Ethyl-10-hydroxycamptothecin[Title/Abstract]) 8573**

**#3 ((((((((UGT1A1 enzyme[MeSH Terms]) OR (UDP glucuronosyltransferase 1A1[Title/Abstract])) OR (phenol UDP-glucuronosyltransferase[Title/Abstract])) OR (1-naphthol UDP-glucuronosyltransferase[Title/Abstract])) OR (phenol GCS transferase[Title/Abstract])) OR (uridine diphospho* glucuronosyltransferase 1A1[Title/Abstract])) OR (UDP-glucuronosyltransferase 1A1[Title/Abstract])) OR (UGT1A[Title/Abstract])) OR (UDP glycosyltransferase 1 family, polypeptide A1[Title/Abstract]) 2675**

**#4 #1 and #2 and #3 178**

**Search Strategy of OVID EMBASE**

1 colorectal tumor.mp. or exp colorectal tumor/ 33378

2 colorectal cancer.mp. or exp colorectal cancer/ 239382

3 colorectal carcinoma.mp. or exp colorectal carcinoma/ 35003

4 exp colon tumor/ or colon* tumor.mp. 352464

5 exp colon cancer/ or colon* cancer.mp. 315014

6 exp colon carcinoma/ or colon* carcinoma.mp. 41344

7 exp rectum tumor/ or Rect* tumor.mp. 283298

8 exp rectum cancer/ or Rect* cancer.mp. 251266

9 exp rectum carcinoma/ or Rect* carcinoma.mp. 16996

10 1 or 2 or 3 or 4 or 5 or 6 or 7 or 8 or 9 432138

11 irinotecan.mp. or exp irinotecan/ 42476

12 camptothecin 11.mp. 47

13 NK012.mp. 39

14 CPT11.mp. 159

15 CPT 11.mp. 5787

16 Camptosar.mp. 965

17 7-Ethyl-10-hydroxycamptothecin.mp. 2417

18 11 or 12 or 13 or 14 or 15 or 16 or 17 43454

19 UGT1A1.mp. or exp glucuronosyltransferase 1A1/ 5028

20 UDP glucuronosyltransferase 1A1.mp. 286

21 exp glucuronosyltransferase/ or 1-naphthol UDP glucuronosyltransferase.mp. 8091

22 uridine diphospho* glucuronosyltransferase 1A1.mp. 16

23 19 or 20 or 21 or 22 11789

24 10 and 18 and 23 702

**Search Strategy of OVID MEDLINE**

1Colorectal Neoplasms.mp. or exp Colorectal Neoplasms/ 216813

2Colorectal tumor.mp. 2183

3Colorectal cancer.mp. 113559

4Colorectal Carcinoma.mp. 14687

5Colonic Neoplasms.mp. or exp Colonic Neoplasms/ 78438

6 Colon* cancer.mp. 54185

7Colon* tumor.mp. 3091

8Rectal Neoplasms.mp. or exp Rectal Neoplasms/ 50302

9Rect* cancer.mp. 26504

10 Rect* tumor.mp. 816

11Rect* Carcinoma.mp. 3961

12Colon* Carcinoma.mp. 12458

13 1 or 2 or 3 or 4 or 5 or 6 or 7 or 8 or 9 or 10 or 11 or 12 274881

14 Irinotecan.mp. or exp Irinotecan/ 11865

15Irrinotecan.mp. 5

16Camptothecin*11.mp. 14342

17 SN*38*11.mp. 390191

18 NK012.mp. 23

19 CPT*11.mp. 18621

20 Camptosar.mp. 180

21 7-Ethyl-10-hydroxycamptothecin.mp. 399

22 14 or 15 or 16 or 17 or 18 or 19 or 20 or 21 421359

23 exp Glucuronosyltransferase/ or UGT1A1.mp. 8735

24 UDP glucuronosyltransferase 1A1.mp. 231

25 1-naphthol UDP glucuronosyltransferase.mp. 7

26 uridine diphospho* glucuronosyltransferase 1A1.mp. 15

27 23 or 24 or 25 or 26 8766

28 13 and 22 and 27 277

**Search Strategy of CENTRAL**

1 MeSH descriptor: [Colorectal Neoplasms] explode all trees 8661

2 (Colorectal tumor?):ti,ab,kw 5216

3 (Colorectal cancer?):ti,ab,kw 15534

4 (Colorectal Carcinoma?):ti,ab,kw 2134

5 MeSH descriptor: [Colonic Neoplasms] explode all trees 1811

6 (Colon* cancer?):ti,ab,kw 10158

7 (Colon* tumor?):ti,ab,kw 4002

8 MeSH descriptor: [Rectal Neoplasms] explode all trees 1904

9 (Rect* cancer?):ti,ab,kw 7028

10 (Rect* tumor?):ti,ab,kw 2992

11 #1 or #2 or #3 or #4 or #5 or #6 or #7 or #8 or #9 or #10 28039

12 MeSH descriptor: [Irinotecan] explode all trees 914

13 (Irrinotecan):ti,ab,kw 6

14 (Camptothecin?11):ti,ab,kw 0

15 (NK012):ti,ab,kw 1

16 (CPT?11):ti,ab,kw 31

17 (Camptosar):ti,ab,kw 29

18 (7-Ethyl-10-hydroxycamptothecin):ti,ab,kw 10

19 #12 or #13 or #14 or #15 or #16 or #17or #18 976

20 MeSH descriptor: [Glucuronosyltransferase] explode all trees 68

21 (UGT1A1):ti,ab,kw 256

22 (UDP glucuronosyltransferase):ti,ab,kw 52

23 (UDP glycosyltransferase 1 family, polypeptide A1):ti,ab,kw 0

24 (uridine diphospho* glucuronosyltransferase 1A1):ti,ab,kw 1

25 #20 or #21 or #22 or #23 or #24 310

26 #11 and #19 and #25 29
